# Techniques for Abdominal Wall Closure after Damage Control Laparotomy: From Temporary Abdominal Closure to Early/Delayed Fascial Closure—A Review

**DOI:** 10.1155/2016/2073260

**Published:** 2015-12-27

**Authors:** Qian Huang, Jieshou Li, Wan-yee Lau

**Affiliations:** ^1^Research Institute of General Surgery, Jinling Hospital, Medical School of Nanjing University, Nanjing 210000, China; ^2^Faculty of Medicine, The Chinese University of Hong Kong, Shatin, Hong Kong

## Abstract

Open abdomen (OA) has been an effective treatment for abdominal catastrophes in traumatic and general surgery. However, management of patients with OA remains a formidable task for surgeons. The central goal of OA is closure of fascial defect as early as is clinically feasible without precipitating abdominal compartment syndrome. Historically, techniques such as packing, mesh, and vacuum-assisted closure have been developed to assist temporary abdominal closure, and techniques such as components separation, mesh-mediated traction, bridging fascial defect with permanent synthetic mesh, or biologic mesh have also been attempted to achieve early primary fascial closure, either alone or in combined use. The objective of this review is to present the challenges of these techniques for OA with a goal of early primary fascial closure, when the patient's physiological condition allows.

## 1. Introduction

Direct pressure has long been used until World War II for hemostasis, especially in patients with coagulopathy. It was abandoned because of recurrent bleeding at pack removal and late infection. In a prospective study carried out from 1968 to 1973, Lucas and Ledgerwood [1] reintroduced the technique and used it on 3 patients in a series of 637 patients with liver injuries. The authors emphasized “open-mindedness and flexibility” in the use of packing. Subsequently, other authors reported on packing for severe liver injuries [2, 3]. As the number of patients in these series was small (4 and 17 patients), the idea of packing was still not considered as a desirable and standard practice then [2, 3].

In 1983, Stone et al. [4] reported their experience on 31 patients who were diagnosed to have major coagulopathy soon after the onset of surgery. In the first 14 patients, the procedure was continued with hemostatic replacement and completion of the operation. The mortality was 93% [1]. In the subsequent 17 patients, the operation was aborted and abdominal tamponade was achieved with sponges. The mortality was 35%. Unfortunately, the operation was considered as a surgical failure as leaving the abdomen “open” was thought to result in intra-abdominal abscess and intestinal fistulas. In 1993, pioneered by Rotondo et al. [5], surgeons began to recognize that patients with major injuries were more likely to die from intraoperative metabolic failure (the vicious triad of coagulopathy, acidosis, and hypothermia) than from failure to complete operative repairs. The need for abbreviated surgery and rapid return to intensive care units (ICU) for aggressive resuscitation were emphasized. Now codified as “damage-control” laparotomy, patients are left with open abdomen (OA) with a planned return to the operating room for definitive surgery. Thus the concept of damage-control surgery (DCS), used more than 100 years ago, has finally been accepted by mainstream surgeons recently.

DCS and OA are major surgical advances in the care of critically ill surgical patients. It is important to recognize that the role for OA is not exclusive to the damage control population. Intra-abdominal hypertension (IAH) and abdominal compartment syndrome (ACS) have been increasingly recognized as contributing factors for mortality [6]. For clarity of discussion, the definitions of the common terms used are in Table 1. The pathophysiology of IAH/ACS represents a continuum along a spectrum. The higher the intra-abdominal pressure is, the more likely it is for ASC to develop with the subsequent pulmonary, cardiovascular, renal, and gastrointestinal dysfunction/failure. Along with improved understanding of the pathophysiology of inflammation, injury response, and IAH/ACS, the use of open abdomen has increased, which prompted the development of the various techniques of temporary abdominal closure (TAC).

## 2. Indications for Open Abdomen and Temporary Abdominal Closure

The abdomen is left open under the following specific circumstances as part of damage control surgery, severe abdominal infection, planned second look operation, and prevention of abdominal compartment syndrome:

 Severe abdominal infection.
 Infected pancreatic necrosis.
 Vascular (e.g., ruptured abdominal aortic aneurysm, hemorrhage).
 Severe trauma.
 Necrotizing infection of abdominal wall.
 Ischemic gut with planned second look laparotomy.
 Damage control surgery.
 Abdominal compartment syndrome.
 Transplantation with size discrepancy between the recipient's abdomen and the graft.

Open management of severe abdominal infection is indicated in patients in whom a single laparotomy cannot effectively control the source of infection, for example, in patients with infected pancreatic necrosis. Leaving the abdomen open enables repeated access to the peritoneal cavity and facilitates repeated debridement of nonviable tissues, peritoneal toilet, and effective drainage. This procedure can be performed either in the operating room or at the bedside in ICU.

The concepts and techniques of DCS have now been extended to elective and emergency surgery. For trauma, the initial abdominal exploration is designed to control hemorrhage and contamination. For nontraumatic cases, these basic principles have also been applied most commonly for control of an infectious source. Hemorrhage can be controlled by ligating, repairing, or shunting injured vessels or packing solid organ or pelvic injuries. Contamination can be controlled by identifying bowel injuries followed by repair, exteriorizing, or stapling guts ends without any attempt for anastomosis.

The use of open abdomen in patients with ischemia bowel is based on the need for a mandatory “second look” operation to assess bowel viability and to resect additional ischemic bowel segments if necessary. Obvious dead bowel should be resected. However, it is not always obvious that the bowel cannot be saved. If there is any question of bowel viability at initial surgery, extensive resection of potentially viable bowel should be avoided and a second look laparotomy should be planned. Bowel viability can be improved with active resuscitation, and avoiding unnecessary extensive resection prevents the occurrence of short bowel syndrome.

An abdominal compartment syndrome (ACS) plays an important pathophysiological role that leads to visceral hypoperfusion and multiple organ failure. After abdominal decompression for established ACS, the reported survival rates ranged from 33 to 100 percent [7, 8]. ACS can occur in patients without any intra-abdominal injuries (secondary ACS). Substantial bowel or retroperitoneal edema can result from massive fluid resuscitations or from systemic inflammatory responses with capillary leak especially after burns, pancreatitis, and sepsis with increasing intra-abdominal pressure (IAP). Organ dysfunction/failure follows. A value of >20 mmHg has been suggested as a target for decompressive laparotomy. Patients with acutely increased intra-abdominal pressures >25 mmHg even without any acute organ dysfunction should also be considered for prophylactic decompressive laparotomy [9, 10]. Once the fascia is opened prophylactically or therapeutically, a decision has to be made as to how to avoid having a high intra-abdominal pressure again.

## 3. Importance of Closing the Abdomen

The central goal in the management of patients after OA is closure of the fascial defect as early as clinically feasible, but without precipitating ACS. The risks associated with OA include derangement of fluid and electrolyte balance, systemic inflammatory response, formation of gastrointestinal fistula, adhesions, infection, intra-abdominal abscesses, and catabolic state (listed in Table 2).

**Figure 1:**
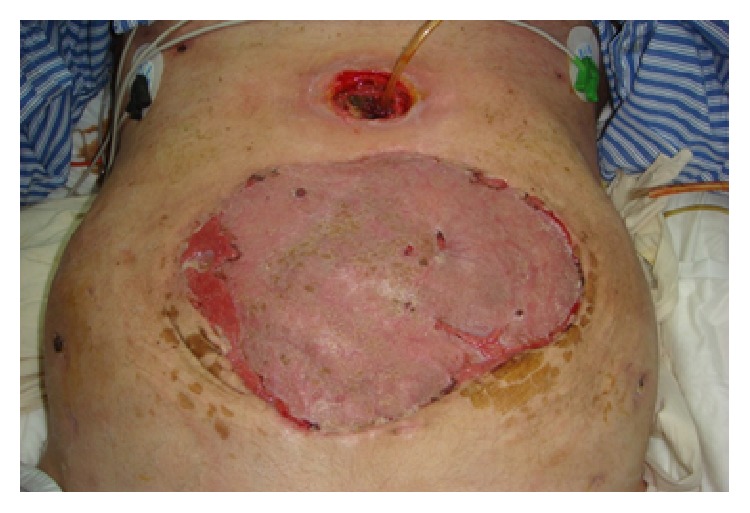
Split-thickness skin graft (STSG) on granulation tissues in open abdomen.

The traditional method to close the fascial defect is to wait for a ventral hernia to develop and then repair the hernia. In this method, the wound is allowed to granulate followed by split-thickness skin grafting (STSG), Figure 1. Six to 12 months later, abdominal wall reconstruction is carried out. However, this approach is associated with the risks associated with OA, physical, and psychosocial stress to the patients and increased cost of medical care. It is recommended that appropriate efforts should be made to attempt definitive fascial closure during the initial hospitalization.

Recent advances in temporary abdominal closure (TAC) techniques in managing open abdomen help to achieve many benefits without incurring much complications. The ideal TAC technique serves as a barrier, thus preventing evisceration and contamination. It assists with evacuation of abdominal fluid and decreases bowel edema. It prevents adhesions and avoids repeated damage to the bowel, fascia, or skin due to exposure. Furthermore, it allows easy access to the abdominal cavity, avoids damage to the fascial edges, prevents fistula formation and allows fistula isolation if present, and prevents abdominal wall retraction while allowing for expansion of abdominal contents to prevent the development of ACS.

## 4. Temporary Abdominal Closure Techniques

### 4.1. Simple Packing

**Figure 2:**
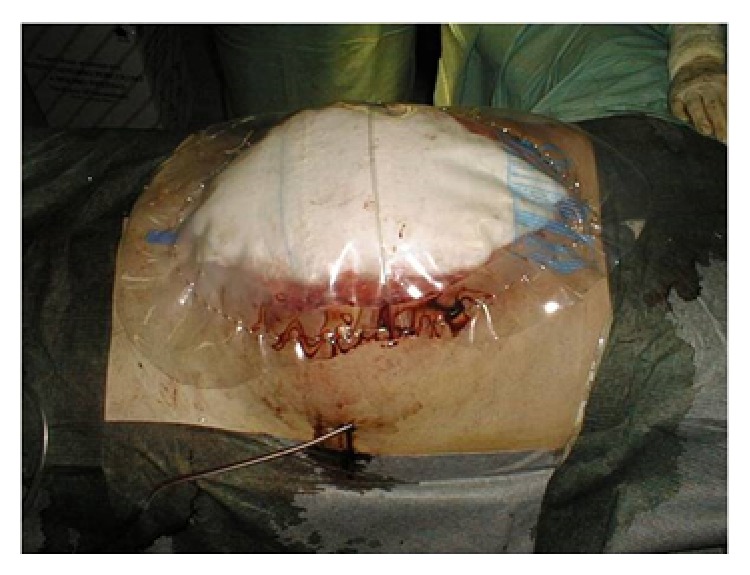
Packing with large saline-soaked gauzes over abdominal contents, followed by a transparent dressing.

Surgical techniques for management of open abdomen have evolved tremendously in the past three decades. Simple packing was the most commonly used technique in the 1980s [11–15]. This method was used to leave the abdomen open for peritoneal drainage in patients with complicated peritonitis or abscess. At the end of the initial operation, nonadherent wet gauzes or hydrophilic dressings were placed directly on top of the abdominal contents, without the use of any sutures (Figure 2). In the intensive care unit, changing of dressings and abdominal lavage were done every day. When compared with the conventional surgical drainage techniques (adequate drainage, wide debridement, and definitive abdomen closure), open packing was associated with less morbidity and mortality [12–14]. Duff and Moffat [12] managed 18 seriously ill patients with abdominal sepsis by leaving the abdomen completely open. The mortality was 39% with six patients who died of sepsis and one from hemorrhage. Davidson and Bradley III [13] reported 17 patients who underwent operations for pancreatic abscess. There was a mortality rate of 55% in patients who underwent sump drainage and 0% in those who underwent open packing. Similar results were found by Maetani and Tobe [14]. In these studies, conclusions were drawn that leaving the abdomen completely open facilitated the widest possible drainage, uncompromised debridement of abdominal wall, and was compatible with good recovery. This technique is preferable to closing an abdominal wall of questionable viability in intraperitoneal sepsis.

From the mid 1980S, surgeons considered “laparotomy” or open packing to be a valuable technique in the management of severe, intra-abdominal sepsis [16–18]. The indications included gastrointestinal perforation, anastomotic dehiscence, abscess, and acute necrotizing pancreatitis. Bailey et al. [18] evaluated laparotomy in patients with severe intra-abdominal infection from colorectal diseases. The mortality was 28.6%. However, the wounds of these patients were left to heal by contraction and granulation and required reconstructive surgery later. Problems with evisceration, fluid and protein loss, and fistula were reported. Although open packing was not an ideal technique, it made surgeons accept that laparotomy was effective in patients with severe intra-abdominal infection.

### 4.2. Skin-Only Closure Techniques

**Figure 3:**
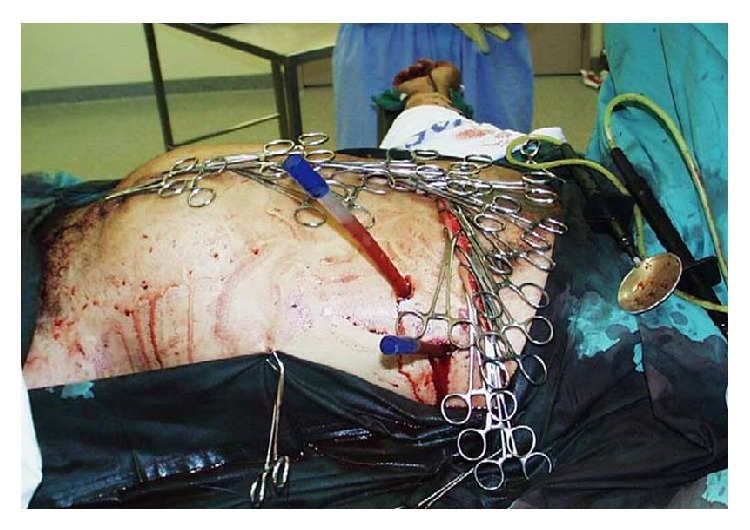
Towel clips were applied to the skin and covered by a plastic drape.

The temporary skin-only closure techniques use the skin to provide some abdominal wall stability with containment of abdominal viscera. These techniques use a series of towel clips or a rapid monofilament running suture [19–21]. The towel clip closure is perhaps the most rapid of the temporary closure techniques. The towel clips are applied to the skin, approximately 1 cm apart. Orienting the handles of all the clips toward the center (up from below and down from above) facilitates coverage of the towel clips by an adhesive plastic drape and minimizes overlying artifact on subsequent radiographs (Figure 3).

Either towel clips or suture closure of the skin is swift, inexpensive, and easily available. The abdominal contents are maintained below the level of the fascia, which minimizes heat and fluid loss. However, as the bursting pressure of the skin is low, both techniques have increased risks of evisceration, injury and loss of skin, infection, and recurrent ACS. Because of the high complication rates, including that of ACS, which varies from 13% to 36% [21], these techniques have largely been abandoned now.

### 4.3. Bogota Bag

**Figure 4:**
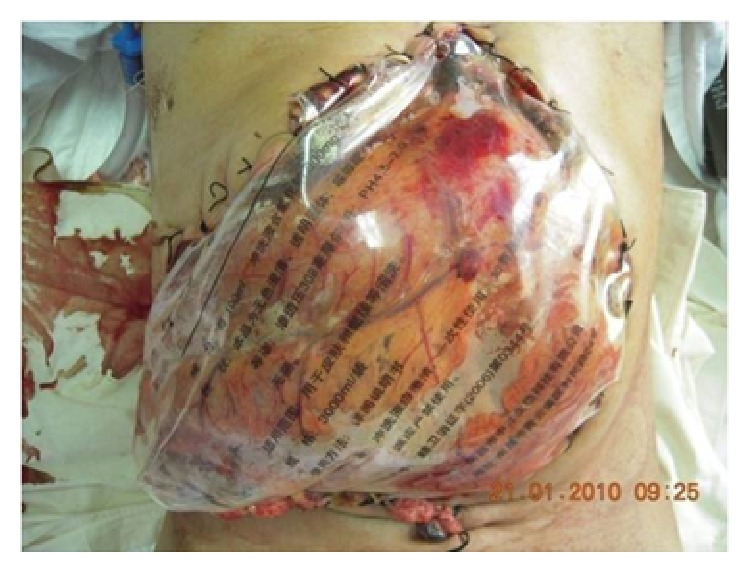
Bogota technique using a presterilized, soft 3-L IV bag.

When skin-only closure is impossible, as is often the case, a temporary plastic Bogota bag sutured to the skin provides an excellent solution for containment. The Bogota bag [22–25], so named by Mattox while observing in Bogota, Colombia, uses a large intravenous (IV) bag to cover the abdominal viscera. After the initial operation, a presterilized, soft 3-L IV bag is cut to an oval shape and stapled with a standard skin stapling device or sutured with monofilament suture to the skin edges of the wound (Figure 4). Sterile, antibiotic soaked towels are placed over the silo, which is then covered with an iodine-impregnated adhesive plastic drape. The wound is inspected and the dressing is changed every 24 hours. IV bag silos may be replaced in the intensive care unit setting using standard sterile surgical techniques and equipment. This is a variation of the silo closure used for repair of gastroschisis and omphalocele. Other alternatives include bowel bag, Steri-Drape, or Silastic cloth.

These materials hold sutures or staples well, help to retain body heat, minimize fluid loss, are quick and easy to apply, and are nonirritating to the underlying viscera. A silo closure may decrease respiratory and renal compromise associated with decrease in intra-abdominal pressure. The Bogota bag closure is much less expensive than any other techniques which are currently available. The flexibility to apply the silo at the bedside also decreases the inherent risks with transferring a critically ill patient from the intensive care unit to the operating room. The technique may be particularly useful for surgeons who encounter severe abdominal trauma in small rural hospitals because life-saving interventions, such as control of bleeding, need to be performed immediately and rapidly before the patients are transferred to a major hospital for definitive treatment. The rates of primary closure range from 12 to 82%, and enterocutaneous fistula rates are generally low, and range from 0 to 14.4% [26–28].

However, Bogota bags do not prevent abdominal wall retraction, and they do not allow effective removal of abdominal fluids. The use of this technique mandates a subsequent procedure to remove the Bogota bag followed by a definitive closure. The subsequent development and repair of large hernias constitute one of the difficult postoperative problems which require future solution. Recently, Joglar et al. [29] reported a modified Bogota bag approach by using dynamic-like retention sutures, which permit preservation of abdominal fascia and decrease the need for a planned ventral hernia repair. Open abdomen with a Bogota bag is associated with a high rate of hospital morbidity and delayed complications. Manterola et al. [30], on evaluating a prospective series of 86 patients who underwent relaparotomy, found that with the most frequent indication of contained laparotomy being intra-abdominal sepsis (60%) the primary fascial closure rate was 39% and the in-hospital mortality rate was 12%. Sixty percent of patients developed a ventral hernia within a follow-up of 48 month.

### 4.4. Mesh

**Figure 5:**
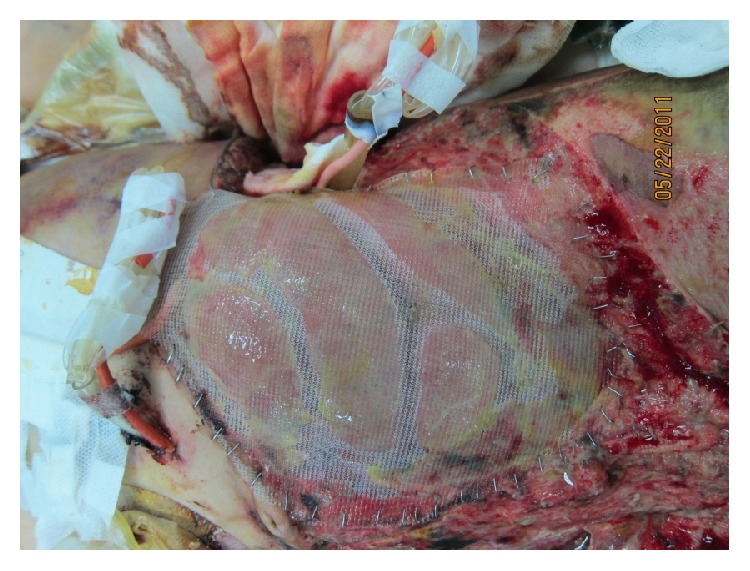
Laparotomy using a 30*∗*30 cm polypropylene mesh sutured to the fascial edges with a running suture.

**Figure 6:**
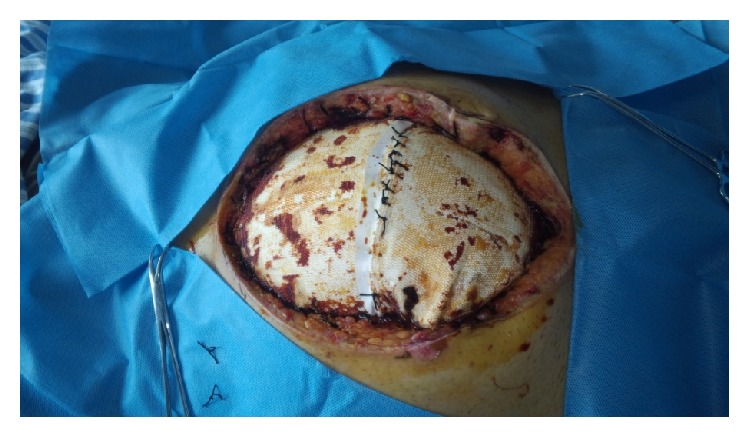
Laparotomy using a polytetrafluoroethylene (ePTFE) to cover the bowels.

The use of absorbable and permanent synthetic repair materials for patients who underwent TAC in open abdomen has been reported [31–45]. Permanent synthetic prostheses, when sutured to the fascial edges, can be used to protect abdominal wall tissues from damages which result from repetitive surgical procedures through the incision, prevent lateral retraction of the fascia, and facilitated reoperation. However, wrinkling secondary to wound contracture, infection, hernia, mesh extrusion, and enterocutaneous fistula are some complications that may be seen. Several permanent synthetic repair materials are available, including the broad classes of macroporous, microporous, and composite materials. When implanting a prosthetic mesh, the surgeon must carefully take into consideration the potential interactions between the host and the prosthetic material. As a result of fibroblast response produced by the rough texture of the mesh, the mesh material becomes incorporated into the host's granulation tissues. In this way, intestinal loops become adherent to the biomaterial to form the first stage in the development of biomaterial-related intestinal fistula. Polypropylene mesh, with its porous structure which is appropriate for growth of microvascular vessels and convenient for topical application of growth factors, was popularly used (Figure 5). Yuan et al. [43] reported that when compared with polyethylene sheet (always Bogota Bag in emergency situations), the microvascular densities, thickness of granulation tissue, and fibroblast counts were higher in the polypropylene mesh group. Although meshes improved the primary closure rates which ranged from 33 to 89%, macroporous repair materials such as polypropylene were associated with a high incidence of 6.6% to 14.7% of fistulas when placed in contact with bowel [31, 32, 35–37, 39]. Some series reported fistula formation in as many as 75% of patients [33]. Recently, Scholtes et al. [44] reported that implantation of nonabsorbable meshes in open abdomen, even for a patient with a contaminated or dirty abdomen, resulted in a reduced incidence of incisional abdominal wall hernia. The overall mortality rate and enterocutaneous fistula formation rate were 8% and 22%, respectively, which were not influenced by the use of mesh. However, the limitations of this study were that it was retrospective in design, and the authors could not exclude selection bias in the indication for mesh implantation. Thus, nonadherent materials should be placed between the intra-abdominal contents and fascia to prevent formation of fistulas and to facilitate future manipulation of the wound. Microporous repair materials such as polytetrafluoroethylene (ePTFE) which resists adherence to tissues may be used over the bowel (Figure 6). The main disadvantage of microporous repair materials is the increased risk of infection. The pore size allows for the colonizing bacteria to evade the host immune cells. The prosthetic then acts as a source of contamination in the wound. Thus, both ePTFE and polypropylene materials should not be placed in a contaminated environment, because of unsuitability and the high complication risk. However, this risk should not be an issue if the material is being used as a temporary closure which will be removed at the time of definitive closure [41].

Absorbable meshes include polyglactin 910 (Vicryl; Ethicon, Somerville, NJ) and polyglycolic acid (Dexon; Davis & Geck, Danbury, CT) [34, 38, 40, 42]. Vicryl comes in a larger size (12*∗*12 inches) but has smaller interstices, which tends to impair drainage. Vicryl also tends to tear at the suture site even with a tapered needle. Dexon, although smaller in size (the largest size is 7 × 9 inches), has large interstices which allow easy passage of a needle. It does not tear and allows for egress of intra-abdominal fluid [35]. The advantages of an absorbable mesh are as follows: it is resistant to infection, pliable, and easier to work with than the currently available permanent meshes. The mesh does not unravel when cut and can be opened repeatedly to provide less traumatic access to the abdominal cavity for repeated drainage procedures. When wound contraction occurs, the mesh can be trimmed to remove any excess material. Both types of absorbable meshes result in less tension on the fascia, thus minimizing the occurrence of necrotizing fasciitis. However, the use of absorbable meshes for TAC has resulted in fistula formation rates which ranged from 5% to 11% and intra-abdominal abscesses [41]. Prichayudh et al. reported enteroatmospheric fistula formation in 15% of patients [42]. For this reason, consideration should be given to place a barrier between an absorbable synthetic material and viscera for temporary closure. Recently, Sutton et al. [45] reported the use of Gore Bio-A mesh in the management of open abdomen. Gore Bio-A mesh has a web of biocompatible synthetic polymers which are gradually absorbed in 6 months. The use of Gore Bio-A is safe, feasible, and cost-effective even within a contaminated field. Unlike the permanent meshes, it facilitates ingrowth of granulation tissues causing the covering to adhere to the wound. When this occurs, reoperation becomes a challenge, and the patient must be treated with split-thickness skin graft and then to undergo a planned ventral hernia repair about a year after the initial operation. Therefore, if an absorbable mesh is used for TAC, care should be taken to remove the material before any rapid overgrowth of granulation tissues.

Absorbable mesh is not designed to be used to approximate the fascial edges serially but it is designed to form a granulation tissue bed for future skin grafting. On the other hand, nonabsorbable meshes can be initially sutured to the abdominal fascia loosely, allowing visceral swelling and thus preventing the development of ACS. As visceral edema resolves, the mesh can then be excised in the medial portion and the two edges resutured to sequentially result in fascial approximation. The use of nonabsorbable meshes improves the primary closure rates, which range from 33 to 89% [46].

### 4.5. Wittmann Patch

Ideally, primary closure should be achieved within 7–10 days, and it should be accomplished in the majority of open abdomens. A study has shown that delayed abdominal fascial closure (DAFC) before 8 days was associated with fewer complications: 12% in those closed before 8 days and 52% in those after 8 days [47]. If primary closure cannot be achieved within 8 days, prevention of fascial retraction and serial closure should be initiated. Most of the early methods to deal with open abdomen are to use the planned ventral hernia approach, whereby an absorbable mesh is placed to bridge the fascial gap. This is followed by skin grafting of the granulating wound. More aggressively attempts to achieve delayed fascial closure using alternative techniques have been reported.

**Figure 7:**
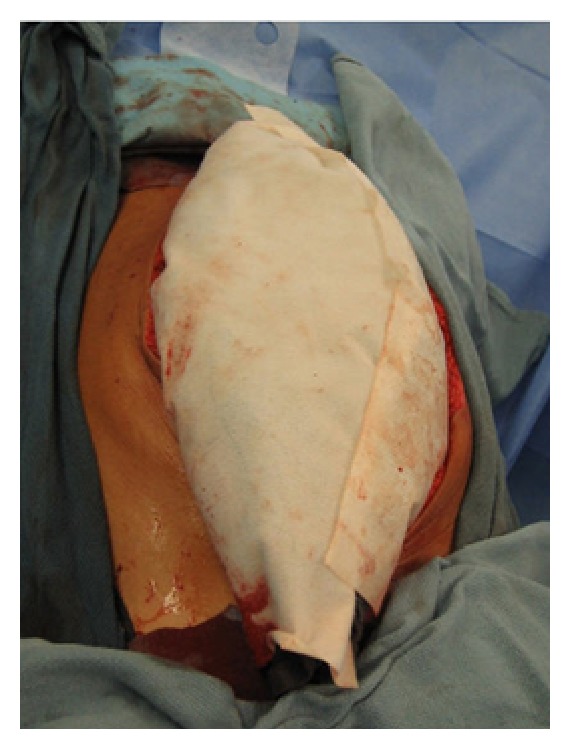
Two sheets of Wittmann Patch which adhere to each other (from [53]; reprinted with permission from Elsevier).

The Wittmann Patch, also called “artificial burr” in reference to the fruit of the plants in the genus* Arctium*, consists of two detachable components—a loop sheet and a closure sheet. Firm pressure causes penetration of the free ends of the “mushrooms” of the closure sheet through the loop sheet, creating a stable configuration between them (Figure 7). The burr can be opened by peeling, but it withstands shearing forces across the laparotomy. Typically, the patch is sequentially tightened every 24–48 h until the fascia is approximately 2–4 cm apart. Then this temporary closure is removed at the final operation and some form of definitive closure is used to close the fascia primarily.

Many methods have been advocated to maintain abdominal integrity and to facilitate fascial approximation, including the use of zippers, slide fasteners, and a Velcro analog. Wittmann et al. compared these several devices for TAC and concluded that the Velcro analog was the most practical option [48]. Aprahamian et al. [49] prospectively studied planned relaparotomy in a series of 20 trauma patients. In 16 survivors who had the Wittmann Patch placed to facilitate abdominal closure, 15 patients (94%) had their fascial closed after removal of the patch. The authors considered that the Wittmann Patch provided a simple method to accommodate the change in abdominal girth and it has not been associated with spontaneous opening. Fantus et al. [50] reported the use of the Wittmann Patch in combination with a nonadherent preventing barrier in 3 trauma patients. Using this modified technique, they were able to achieve complete abdominal fascial closure in all the patients over the last 22 months. No patient had a large, granulating open abdomen. There were no enterocutaneous fistulae and no massive abdominal wall hernia. Several studies reported similar results and showed that the use of the Wittmann Patch achieved a high rate of delayed fascial closure in severe trauma patients [51, 52]. Keramati et al. also reported [53] that in burn patients with abdominal compartment syndrome, the survivors who received Wittmann Patch subsequently underwent primary abdominal closure, with no evidence of ventral hernia on long-term follow-up.

The standard fascial closure using a running suture is safe, and it can be accomplished in 10–15 min. Less time is necessary when using mesh, zippers, slide fasteners, or Wittmann Patch. The mesh, zippers, or Wittmann Patch permits rapid and safe reentry into the abdomen on reexploration, and if an additional laparotomy is necessary in the future, permit a rapid closure. Opening and closure of the Wittmann Patch take only seconds. The added advantages are that it accommodates a decrease in abdominal distension, while slide fasteners and zippers must be removed and new materials are inserted as edema decreases. In mesh devices excess can be excised to accommodate decreases in edema. However, they must be resutured to facilitate future abdominal closure. Other advantages of the Wittmann Patch technique include a gradual approximation of fascia, ease of reexploration, and prevention of loss of abdominal domain. For this reason, this method enjoys popularity and it results in good overall outcomes. The rate of primary closure for the Wittmann Patch ranges from 78 to 100%. The rate of overall complication remains relatively low, and the fistula rate is 0–4.2% [51–53]. The Wittmann Patch technique is more costly and requires suturing to the abdominal fascia, which may increase the risk of fascial trauma and necrosis, and future incisional hernias may develop. Finally, this technique does not effectively evacuate peritoneal fluid, and abdominal wound drainage may become an issue.

### 4.6. Vacuum-Assisted Closure

In the management of OA, the aforementioned methods often need frequent and time-consuming changing of dressing, intensive nursing, and prolonged treatment before achieving final definitive wound closure, all of which may severely impact quality of life. Moreover, these methods do not offer a built-in drainage system to drain peritoneal fluid. The management of the “open abdomen” using negative pressure therapy is not a new concept. Negative pressure therapy (NPT) has been shown to increase local blood perfusion and nutrient delivery to the wound, accelerate growth of granulation tissues, and decrease wound bacterial concentrations. It also reduces bowel edema and the application of mechanical stress to the wound accelerates cellular proliferation and angiogenesis. The negative pressure therapy, by the principle of reverse tissue expansion in the wound, brings together the wound edges [54]. It has been used to improve skin graft survival, treat acute and chronic wounds, pressure sores, venous ulcers, and diabetic foot wounds. Its use in temporary closure for patients with open abdomen has been investigated by surgeons.

**Figure 8:**
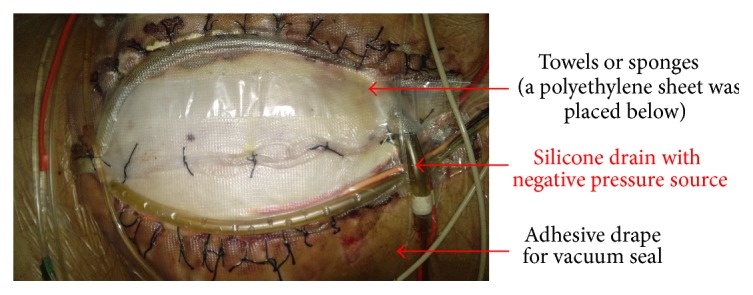
The component layers of the vacuum pack dressings.

Vacuum-assisted closure (VAC) originated with Barker's group in Chattanooga. In 1995, they reported this technique using the term “vacuum pack” [55]. The pack consists of four component layers (Figure 8). The first layer is a perforated polyethylene sheet that is placed beneath the peritoneum of the abdominal wall. This material provides some protection to the viscera, and the nonadherent nature of the polyethylene prevents adhesion of the viscera to itself and to the under surface of the abdominal wall. As a consequence the chances of damage to the viscera on repeat exploration decreases and it allows the abdominal wall to be advanced toward the mid-line. The perforations allow peritoneal fluid to be carried away through the vacuum system, keeping the wound dry. This first layer is the most important to result in a low rate of vacuum-pack-related complications. The second layer consists of suction drains and compressible material, either sterile surgical towels or polyurethane foam. The edges of the towel or sponges are positioned between the perforated polyethylene sheet and the parietal peritoneum of the abdominal wall. Placement of the towel/sponge edges below the peritoneum helps to prevent the viscera from protruding through the abdominal wall defect. When a negative pressure is applied to the dressing, these materials become semirigid, thus providing additional protection and preventing fascial retraction by creating a constant medial tension on the fascia without suture. The third layer consists of silicone drains placed above the towels/sponges and serve as a negative pressure source and a means of controlling egress of intra-abdominal fluid. The drains are connected to a negative pressure source of 100 to 150 mmHg. The fourth layer is an adhesive sheet which serves to cover the skin surrounding the wound and complete the vacuum seal. The dressing is maintained intact under suction until reexploration. Several studies have reported the success use of the vacuum pack dressings with primary fascial closure rates ranging from 35 to 92% and fistula rates ranging from 0 to 15% [55–60].

The two most commonly used negative-pressure dressings systems are the V.A.C. Abdominal Dressing System and ABThera System from KCI. The V.A.C. Abdominal Dressing System consists of an inner plastic-encased sponge designed to be in direct contact with the viscera. The plastic interface protects the bowel, prevents adhesion formation, and is perforated to allow passage of fluids. Next, a macroporous “black” sponge is then applied over the inner layer and it is in contact with the fascia. This sponge can be held in place with skin staples to approximate the skin edges if desired. This layer is then covered with an adhesive occlusive dressing; and a suction drainage device is applied to the superficial foam layer for evacuation. The perforated plastic drape can be placed directly on the viscera and the microporous “white” sponge placed on the plastic followed by the black sponge; alternatively, black sponge can be used for the entire dressing. Many studies have been published to introduce this technique which resulted in high fascial closure rates [61–67]. The ABThera System uses the same technique with improved refinements [68, 69]. It consists of a large visceral protective layer which includes a polyurethane film-covered central foam structure with six arms of polyurethane foam extending from the center to envelop the viscera by extending deep into the paracolic gutters and drains fluid from the gutters, the pelvis, and between loops of bowel. This is colloquially known as the “spider drape.” The inner sponge extensions that extend to the ends of the plastic sheet facilitate effective evacuation of peritoneal fluid. Other parts of the system include an oval-shaped sponge that fits on top of the exposed viscera, to occupy the space between the two edges of the open abdominal wall, a large adhesive drape to create a perfect airtight seal, and a connecting pad that allows for negative suction.

Fitzgerald et al. [69] reported their experiences with the first use of the ABThera System on a patient with acute pancreatitis which required emergency decompressive laparotomy for abdominal compartment syndrome (ACS). The patient was successfully managed by laparotomy and the ABThera System and eventually achieved restoration of gastrointestinal continuity 383 days after admission.

These systems effectively perform the goals of expanding the abdominal cavity, protecting the viscera from heat and evaporative losses, controlling, and quantifying peritoneal fluid and actively removes potentially detrimental contaminated fluid from deep within the abdomen. Early abdominal fascial closure before 8 days has been shown to be associated with fewer complications. However, these systems have the ability to achieve primary facial closure by extending the timing of abdominal closure to be beyond 7 days, generally to 20–40 days [61, 66, 67]. Studies have also shown that these systems are associated with significantly higher 30-day primary fascial closure rates and lower 30-day all-cause mortality rates among patients who require an open abdomen for at least 48 h. Primary fascial closure rates utilizing these systems range from 33 to 100% (average 57.8%). Fistula rates range from 0 to 15% (average 7%), which are similar to the vacuum pack system [70]. Recently, Cheatham et al. [71] conducted a prospective, observational, open-label study to evaluate two TAC techniques in patients requiring open abdomen management. This study found that by comparing with the vacuum pack dressings group, the ABThera System was associated with a significantly higher 30-day primary fascial closure rate and a lower 30-day all-cause mortality rate. The disadvantages include high costs of the commercial dressings and inability to place the system over protruding viscera.

### 4.7. Vacuum-Assisted Wound Closure and Mesh-Mediated Fascial Traction

The vacuum-assisted closure technique for handling open abdomen has improved the care and increased the possibility of fascial closure in the open abdomen. Unfortunately, occasional failures with this technique occur in patients with severe visceral swelling which requires long treatment periods with open abdomen. In 2006, Fantus et al. [72] reported in trauma patients a new technique which combined the vacuum pack technique with continuous medial fascial traction through a Wittmann patch sutured to the edges of the fascia, thus leading to a higher incidence of fascia-to-fascia abdominal wall closure. Using a polypropylene mesh instead of a Wittmann patch, Petersson and colleagues [73] described a novel technique for late closure of open abdomen on seven patients (5 with ruptured abdominal aortic aneurysm, RAAA). They named the procedure vacuum-assisted wound closure and mesh-mediated fascial traction (VAWCM). The majority of patients in the study were old and had renal failure, and the extreme visceral swelling in these patients required treatment with OA for longer time periods. However, all these patients had delayed primary fascial closure even after a median of 32 days, indicating that this combination of techniques offers an advantage. This study was criticized for small patient sample size and retrospective design. Acosta et al. and Rasilainen et al. conducted multicentre prospective and randomized controlled trials on this combination of techniques. The delayed fascial closure rate ranged from 78% to 89% and enteroatmospheric fistula rate ranged from 7% to 12%, although some patients developed intestinal ischemia [74, 75]. Moreover, a study by Sörelius et al. provided further evidence that VAWCM facilitated abdominal wall closure in a selected subgroup of patients with open abdomen after abdominal aortic aneurysm (AAA) repair [76]. The fascial closure rate was higher and the related complication rate was lower than the VAC treatment alone or the mesh traction technique alone. In our practice [77], the VAWCM technique can be successfully used in septic patients even when the wound was complex and/or contaminated. The complete fascial closure rate was 60% and the technique-related complications were few. Recently, Bjarnason et al. [78] addressed the long-term results after OA with a primary focus on hernia development after successful delayed primary fascial closure within the first year [74]. In this study, among the 64 survivors who received delayed primary fascial closure, 36% patients had a clinically detectable hernia and 30% of patients had hernias that were detected on CT or at laparotomy. The authors concluded that the incidence of incisional hernia 1 year after OA treated with VAWCM was high. However, most of these hernias were small and asymptomatic, with few requiring surgical repair, which are completely different from the giant planned ventral hernias of the past.

**Figure 9:**
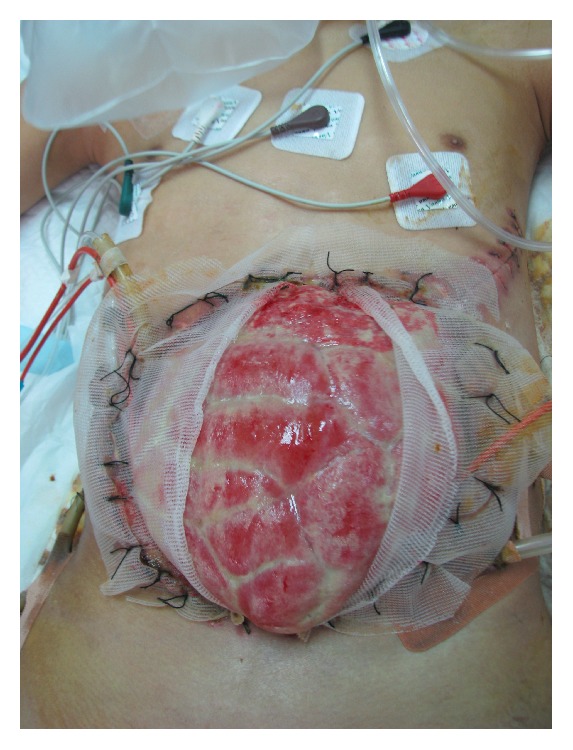
A polypropylene mesh was sutured to the fascial edges using a running 0 monofilament suture.

**Figure 10:**
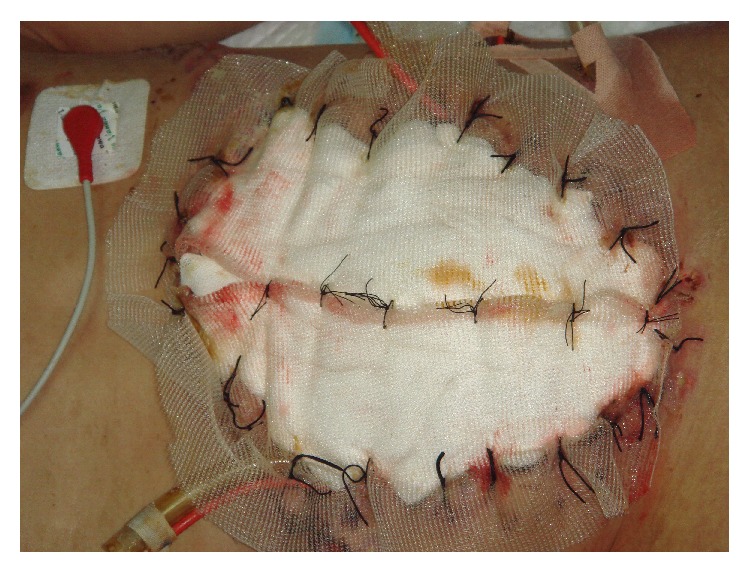
The moist laparotomy pads protected the fascia and the subcutaneous tissues.

The principle using VAWCM as a technique for temporary abdominal closure after laparotomy has been described [73–77]. In brief, in a patient whose abdomen was left open, a sterile perforated plastic sheet was placed intra-abdominally to cover the viscera and then an oval-shaped polypropylene mesh is sutured to the fascial edges using a running 0 monofilament suture. The plastic sheet is covered with moist laparotomy pads, to protect the fascia and the subcutaneous tissues. A sterile gauze is placed over the pads and two silicone drainage tubes are brought in caudally through the skin over the gauze. The drains are covered with a layer of dry laparotomy pads and the wound is sealed with adhesive plastic dressings. The drains are linked to a suction device under continuous topical negative pressure (100–150 mmHg) (Figures 9 and 10). After 2-3 days the possibility to close the abdomen is evaluated. If possible, the abdominal wall is closed. Otherwise, the mesh is cut in the midline, the inner plastic sheet and gauze are changed, and the mesh is tightened by suturing it in the midline using a running 0 monofilament suture, while the viscera is kept from protruding by putting some tension on the abdominal wall. This temporary abdominal closure system is changed every 2-3 days. Abdominal closure is considered when a separation of the fascial edges remains from 3 to 5 cm, with only weak tension assessed by pulling the fascial edges towards the midline. The mesh is then removed, and the fascia is closed. This is followed by skin closure.

The main advantage of the VAWCM technique is that a combination of techniques works in a synergistic way to facilitate closure of open abdomen. Another advantage is the possibility of cleansing the entire abdominal cavity during the period of treatment, when the entire length of the incision is accessible until the fascia is finally closed. Furthermore, the VAWCM technique also allows the abdominal wall to move freely toward the midline at every dressing change, without interfering with the adhesions between the bowel and the abdominal wall. A disadvantage of the VAWCM therapy is the need for trained personnel to do the dressing changes and fascial traction.

## 5. Fascial Bridge Techniques for Primary Fascial Closure

The primary goal of progressive reduction of the fascial defect is to achieve a definitive closure of open abdomen within the initial hospitalization. Closure of the fascia should be performed without undue tension because excessive tension on fascial closure can result in increased IAP, ventral hernia, or fascial dehiscence. As described above, through the appropriate use of the TAC techniques, patients with open abdomen can undergo multiple reoperations with progressive and final closure of the fascial defect. However, patients who have ongoing intra-abdominal infection, visceral edema, loss of abdominal domain or fascia, or complicated wound problems; delayed abdominal fascial closure (DAFC) may not be possible. Under such conditions, the limited available surgical options include performing an acute abdominal wall reconstruction using the component separation technique; bridge repair of fascial defect using synthetic/prosthetic mesh or biologic mesh; or a planned ventral hernia.

Component separation, first described by Ramirez et al. in 1990 [79], reconstructs the midline defect with an innervated advancement of muscle and fascia. The technique consists of the following: (1) the anterior abdominal wall skin flaps are developed and dissected from the anterior superior iliac spines to the chest wall, (2) the aponeurosis of the external oblique muscle is divided lateral to the semilunar line at the level of the xiphoid, (3) the external oblique is feed, which will allow the rectus myofascial component to be mobilized medially, and (4) the midline is sutured together. This technique facilitates release of the lateral oblique muscles and is helpful in closing persistent fascial defects. Bilateral advancement yields enough mobility to close defects of 10 cm in the epigastrium, 20 cm at the umbilicus, and 6 cm at the suprapubic level. However, its use for acute definitive closure in the setting of open abdomen has not been well studied. In severe intra-abdominal sepsis, visceral and abdominal wall edema, and ongoing systemic sepsis, component separation is not advisable. There have been no published clinical data or controlled clinical trials reporting the use of component separation in primary closure after OA. In the opinion of the Open Abdomen Advisory Panel (OAAP), full component separation should not be used to promote fascial closure in patients with OA during the initial hospitalization but should only consider the technique to be an “elective” reconstructive technique [80]. Some surgeons do occasionally mobilize components of the abdominal wall such as undermining the skin and release the lateral oblique muscles to close the abdominal wall defect acutely. It should be noted that such procedures do not constitute a full component separation.

### 5.1. Fascial Bridge Using Prosthetic Mesh

Under the situation that the abdominal fascia does not gather together, the first choice of primary fascial closure is fascial bridge with a prosthetic mesh or a biological mesh, or the other option is a planned ventral hernia. The ideal permanent prosthetic mesh for abdominal fascial bridge should have the following properties: chemical inertness, no allergic or inflammatory reaction, ability to resist mechanical stress, ability to be sterilized, lack of physical modification by body tissues, lack of carcinogenicity, convenience for clinical use, and inexpensiveness [25]. In the early periods, nonabsorbable meshes, such as polypropylene, polytetrafluoroethylene, and polyester products which have some but not all these properties, were commonly used for delayed abdominal fascial closure (DAFC) in the management of patients with OA. However, the risk of wound infection, enterocutaneous fistula, and recurrent herniation, which may even develop years after the original procedure, hindered the use of nonabsorbable mesh. Voyles et al. [81] used polypropylene mesh in a large series of patients to bridge repair in acute replacement of full-thickness abdominal wall. It was noted that polypropylene mesh was highly effective in the early restoration of abdominal wall continuity. However, significant long-term problems such as mesh extrusion and/or enteric fistulae developed. The causes of enterocutaneous fistulae include prior bowel desiccation, adherence of bowel to mesh, and adherence of bowel to exposed fascial edges. Once bacterial colonization or infection happened, the prosthetic mesh may act as a chronic source of contamination [82]. Fansler et al. [83] reported similar experience. In 26 critically ill or injured patients requiring celiotomy, polypropylene mesh was used to bridge the fascial defect. Even though the mesh protected the abdominal viscera from damage resulting from repetitive surgical procedures through the same incision, they found the mesh created a 50% enterocutaneous fistula rate. Other long-term complications such as wrinkling secondary to wound contracture, infection, hernia, and mesh extrusion were also seen. The use of permanent prosthetic mesh has been abandoned because of the high rates of complications. Nowadays, prosthetic mesh was mostly used in elective cases but not after trauma or abdominal catastrophes. Tension-free repair of large ventral hernias with prosthetic mesh was associated with hernia recurrence rates which ranged from 2% to 18% and complication rates which ranged from 10% to 17% [84–86]. The association of prosthetic mesh with bacterial colonization is well known, which has been reported to be up to 6.8% [87, 88], even in the absence of contamination. Recently, Dietz et al. [89] presented a four-stage procedure to bridge repair the fascial defect of patients with OA through application of a two-component mesh of polypropylene in combination with polyglycolic acid (PGA, absorbable mesh). 17/19 (89.5%) patients succeeded in abdominal wall closure, except in two patients (9.5%) who died during hospitalization. This indicated that synthetic meshes still represent an important alternative to achieve fascial closure in patients with OA. However, long-term follow-up is still lacking.

### 5.2. Biologic Mesh

Prosthetic mesh allows for a tension-free repair of the fascial defect. Unfortunately, it is associated with a completely different set of problems. In addition, it does not bring any of the basic wound healing units (e.g., glycosaminoglycans, fibronectin) into the wound field. The mesh becomes only minimally integrated in the final wound and it is never truly an integrated implant. Several approaches have been developed in an attempt to address these problems. Mathes et al. reported that reimplantation of a prosthetic into an already contaminated field or skin at risk for breakdown had a very high rate of reinfection [90]. Other methods have been attempted to repair the defects without using a mesh. Local flaps, pedicle or free flaps, have been utilized to provide additional soft tissue coverage and the necessary ingredients for wound healing. However, it cannot be applied universally even if successful in some patients. The fascial defect may be too large for these tissue flaps to cover. Autologous tissue is neither always available nor is it free of donor morbidity. Postoperative complications and reherniation still are troublesome problems with rates ranging from 0% to 43% and 8% to 32%, respectively [91, 92]. Therefore, an ideal prosthesis is one that augments the body's natural efforts to heal, provides structural support, allows for ingrowth, and is eventually replaced or fully integrated. Many of these characteristics are found in acellular dermal matrix (ADM).

ADM is a biologic material derived from a donor source—which in most cases is human cadaveric, porcine, or bovine in origin. Chemical and physical processing removes all cellular components of the dermis while preserving the extracellular matrix and basement membrane components. This results in a sheet consisting of extracellular material that acts as a signal for fibroblast incorporation, collagen deposition, and collagen maturation. The extracellular matrix stays intact and is gradually revascularized and remodeled into autologous tissue while maintaining its structural integrity. Early revascularization of the graft is thought to enhance resistance to infection and contamination. This unique ability also carries ADM to become integrated into the native tissue, which aids in wound strength and offers a more biocompatible solution. In addition, with absence of a permanent prosthetic mesh at the repair site, ADM also shows excellent mechanical properties, such as tensile strength, plasticity, and flexibility [93]. Since 2003, ADM has been introduced for abdominal wall reconstruction, with suggestions that it has improved capacity to integrate with surrounding tissues, with less inclination towards infection, erosion, extrusion, adhesion formation, and rejection when compared with synthetic materials. In addition, there have been reports of successful reconstructions of large, complex abdominal wall defects even when placed directly over viscera and when the operative field had been irradiated and/or contaminated with bacteria [94]. However, the disadvantages of ADMs have surfaced with increasing use. Specifically, one of the most prominent concern with ADM is that it stretches over time, leading to abdominal wall laxity and recurrent hernia which ranged widely from 0% to 80% [95]. The laxity and/or recurrence, however, are likely a function of both the high content of elastin in the dermal matrix and improvement in the underlying bowel edema over time. The recurrence rate is also associated with the ADM location (underlay/inlay, overlay/onlay), type of fascial repair (reinforced/bridged repair), and surgical indication (open abdomen/tumour/recurrent hernia).

**Figure 11:**
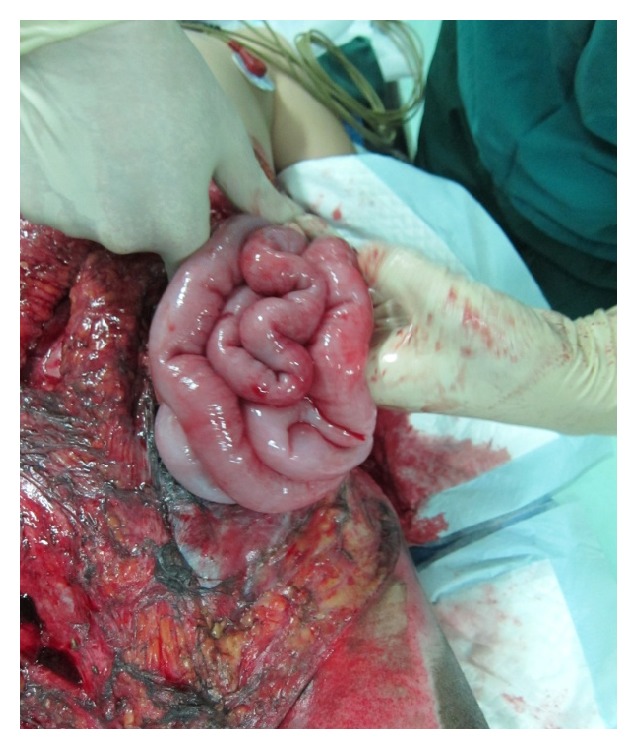
Abdominal dehiscence with exposed bowel in a patient treated with open abdomen.

**Figure 12:**
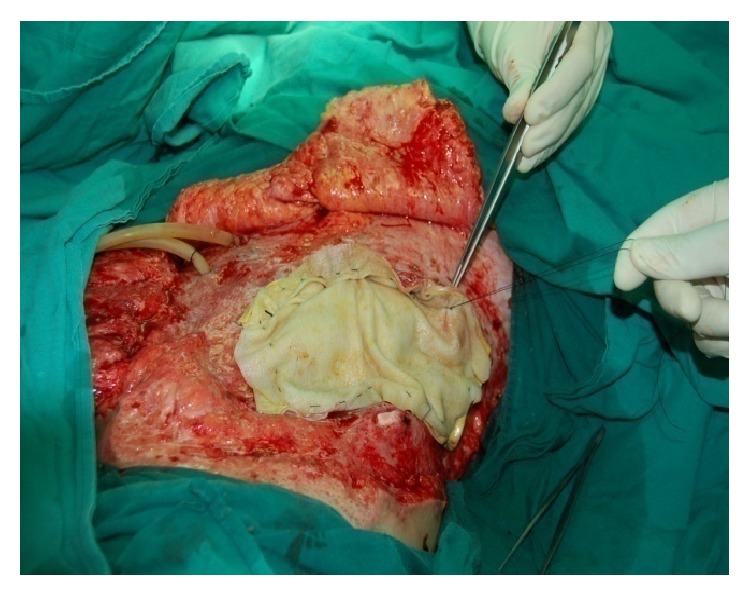
ADM mesh sutured in place over the exposed bowel.

Although ADM has been used extensively as an option to repair hernia defects, even in wounds with complex and contaminated abdominal defects, it is important to recognize that this material is still in the early stages of use in the management of open abdomen (Figures 11-12). Several studies have described the use of ADM as a fascial bridge after open abdomen which may represent a definitive repair for patients with OA. Guy et al. [96] first described a definitive closure technique with a single operation using commercially available ADM as a fascial substitute. ADM materials were used in 9 patients with abdominal compartment syndrome in that trauma center. The fascial defects of the patients were closed on the ninth postoperative day (range 3 to 30 days) and the patients were discharged home on average 8 days (range 5 to 29 days) after the abdominal closure. Complications were few and developed in 3 (33%) patients which included flap hematoma, wound infection, and recurrent hernia. No fistulas developed. A retrospective review of 37 patients with OA reported excellent outcomes. The placement of ADM to bridge repairs the defects that could not be closed primarily after application of negative pressure therapy closed for an average of 21.8 days. No hernia, fistulas, or other complications were reported with a complete follow-up at 30 days and a longest follow-up of 3 years [97]. Conversely, de Moya et al. [98] reported their experience in the use of ADM in trauma patients with large open abdominal wounds and assessed the long-term outcomes. In 10 patients enrolled during a 1-year period, the 30-day follow-up showed no recurrence in 100% of patients. However, follow-up at the end of 1 year demonstrated significant laxity or recurrent hernia or both in 100% of patients. This is the limitation which needs to be addressed surgically. Singh et al. [99] reported in a retrospective review 10 liver transplant recipients with open abdomen treated with ADM. The median follow-up was 10 months with no incidence of evisceration or hernia. Shinall Jr. et al. [100] examined the benefits in children of early fascial closure of open abdomen using ADM. In 5 consecutive children sustaining intra-abdominal catastrophe and managed with damage control celiotomy, ADM was sewn in place as a fascial substitute. After definitive closure, particularly, closure of the skin and subcutaneous tissue over ADM, no patient developed a ventral hernia. Chuo and Thomas [101] described similar results in one aged female with OA using a Permacol mesh (a porcine dermis-derived biomaterial) combined with topical negative pressure therapy. The abdominal dehiscence and exposed bowel of the patient who was admitted with a large bowel perforation were extremely well managed by the technique of mesh application. Permacol mesh appeared to lose its superficial surface at about 4 weeks with the deeper layer incorporating into the underlying wound bed. When she was reviewed later at a 12-month follow-up, examination of the anterior abdominal wall did not reveal any herniation. These available literatures supported the use of ADM as a fascial bridge in the setting of the unclosable abdomen. Yet there is still lack of prospective randomized controlled trials demonstrating the superiority of this technique.

## 6. Summary

The open abdomen technique is one of the greatest advances in recent decades and has become a common procedure in both traumatic and general surgery. One of the primary goals of OA treatment is closure of the fascial defect as quickly as is clinically feasible without increasing intra-abdominal pressure during the initial hospitalization. Multiple techniques have been shown useful in improving the primary abdominal fascial closure rate. There have been few high-quality comparative data to help clinicians to choose among the available techniques. It is the responsibility of the clinicians to apply the basic principles of OA management judiciously to obtain the most benefit for their patients.

## Figures and Tables

**Table 1:** Definitions of common terms.

Terms	Definitions
Intra-abdominal hypertension	Sustained or repeated pathologic elevation of IAP 12 mmHg or greater
ACS (abdominal compartment syndrome)	Sustained IAP greater than 20 mmHg with evidence of new-onset end organ dysfunction or failure
Primary ACS	ACS occurring in the context of abdominal injury
Secondary ACS	ACS occurring without the presence of intra-abdominal injury
DCS (damage control surgery)	Abbreviated laparotomy performed on a critically ill patient aiming to stop major hemorrhage and/or control infectious sources before stabilization of the patient in a critical care unit
Early fascial closure	Fascia-to-fascia closure of abdominal defect with or without prosthetic repair material within 7 days of open abdomen
Delayed fascial closure	Fascia-to-fascia closure after 8 days of open abdomen, usually within the initial hospitalization
Planned ventral hernia	An open abdominal wound that is allowed to granulate and covered with a skin graft before patient discharge from hospital with an intention to perform definitive repair in 6 to 12 months

**Table 2:** Risks associated with open abdomen.

Local effects	Systemic effects
Gastrointestinal fistula formation	Systemic inflammatory response
Intra-abdominal abscesses	Derangement of fluid and electrolyte balance
Abdominal infection	Sepsis
Adhesions causing bowel obstruction	Capillary leak
Adhesions precluding subsequent surgery and/or primary closure	Catabolic state
Fascial retraction	—
